# Feasibility of a 12-Month Exercise Intervention in Postsurgical Colorectal Cancer Patients

**DOI:** 10.1155/2023/4488334

**Published:** 2023-01-20

**Authors:** Melanie Heitkamp, Bianca Spanier, Pia von Korn, Sebastian Knapp, Claudia Groß, Bernhard Haller, Martin Halle

**Affiliations:** ^1^Department of Prevention and Sports Medicine, University Hospital “Klinikum Rechts der Isar”, Technical University of Munich (TUM), Munich, Germany; ^2^Institute of Medical Informatics, Statistics and Epidemiology, Technical University of Munich (TUM), Munich, Germany; ^3^German Center for Cardiovascular Research (Deutsches Zentrum für Herzkreislaufforschung, DZHK), Partner Site Munich Heart Alliance, Munich, Germany

## Abstract

**Background:**

Extensive physical activity (PA; ≥18 MET*∗*h/week, MET metabolic equivalent of tasks hours) postcancer diagnosis has shown favorable effects on colorectal cancer disease-free survival. However, the feasibility of introducing this high volume of PA in this patient group is unclear. Therefore, the aim of the F-PROTECT study was to evaluate the feasibility of extensive and prolonged PA (≥18 MET*∗*h/week over 12 months) in colorectal cancer patients with the primary objectives to (1) recruit 50 patients within 12 months and (2) reach an attendance rate of ≥70%.

**Methods:**

Single-armed, bicentric, prospective intervention study in colorectal cancer patients (≤80 years; UICC II/III Union for International Cancer Control) after histopathological confirmed *R*0-resection who were consecutively recruited from visceral surgery units of 10 clinics in Germany. Recruitment rates were calculated using screening logs. Intervention was a 12-month endurance-focused exercise program with supervised and home-based training. Attendance rates defined as ≥70% participation in training sessions were calculated by training diaries.

**Results:**

Out of 521 patients who were screened for eligibility, 50 (23 female; 59 ± 10 years, UICC 44% II, 56% III; adjuvant chemotherapy 60%) were recruited within 15 months. Mean duration between surgery and first training was 103 ± 57 days. Training attendance rate was 64% (including 9 dropouts). Six (12%) participants reached ≥18 MET*∗*h/week in ≥70% of training sessions between 4–12 months. 28 adverse events (*n* = 9 serious) occurred, however, were not assessed as training related.

**Conclusions:**

The present intervention involving a combination of supervised and home-based exercise training in postsurgical colorectal cancer patients was not feasible. Strategies specifically designed for this patient group must be developed and investigated to motivate long-term PA. *Registration*. The study was prospectively registered at clinicaltrials.gov (NCT01991847).

## 1. Introduction

Colorectal cancer (CRC) is the third most common cancer worldwide and is responsible for about 10% of all cancer deaths [1]. As a result of optimized therapy methods, the relative 5-year survival rate of CRC has improved over the last decades and is above 60% in most European countries [2]. One cornerstone of the posttreatment management in CRC survivors is a healthy lifestyle. Physical activity (PA) intervention trials have been shown to improve the quality of life, PA level, and exercise capacity in CRC survivors [3]. Furthermore, there is growing evidence from cohort studies that PA even has the potential to improve overall and CRC-specific mortality in CRC survivors. A current meta-analysis over 13 studies investigated the association between PA and prognosis in 19.135 nonmetastatic CRC patients after a curative resection. Compared to a PA level of <3 metabolic equivalent task hours per week [4] (MET*∗*h/week), PA levels of ≥18 MET*∗*h/week were associated with a 36% reduction in overall mortality and a 34% increase in cancer-specific survival [5]. Accordingly, lifestyle intervention programs are required for CRC patients after primary treatment to increase their PA level. However, difficulties in recruiting CRC patients for lifestyle interventions must be considered [6]. It has been shown that adherence to PA recommendations is low in cancer survivors, particularly in CRC [7]. In addition, it is unclear whether an extensive exercise training volume of ≥18 MET*∗*h/week can be maintained over a prolonged period of time in this population, as most previous exercise intervention studies have examined only short intervention periods lasting maximum 6 months [3, 8, 9].

Therefore, the feasibility of the potential role of tertiary prevention by exercise in CRC therapy study (F-PROTECT) was initiated to investigate the feasibility of an extensive exercise intervention over 12 months in postsurgical CRC patients. The main objectives were to (1) recruit 50 patients within 12 months and (2) reach an attendance rate (defined as ≥70% participation in training sessions) ≥70%.

## 2. Methods

### 2.1. Study Design

The F-PROTECT study is a prospective, single-armed, bicentric feasibility study, which was conducted in Munich and Ingolstadt (German cities) between January 2014 and April 2016. The study has been carried out in accordance with the Code of Ethics of the World Medical Association (Declaration of Helsinki). The study was approved by the ethics committee of the Faculty of Medicine of the Technical University of Munich, Germany (5888/13). The study was prospectively registered at clinicaltrials.gov (NCT01991847).

### 2.2. Recruitment

The aim was to recruit 50 patients within 12 months. Patients were consecutively enrolled from visceral surgery units of ten clinics (nine in the metropolitan area of Munich and one in the smaller city of Ingolstadt). They were asked to participate in the study during their inpatient stay before or after surgery by clinical staff (physicians, study nurse) or study investigators or contacted by letter after their inpatient stay. The time between recruitment and surgery had to be less than six months. Recruitment was continuously documented in screening logs for each clinic. Written informed consent was obtained from each patient before study enrolment. The initially defined eligibility criteria were age <75 years, histologically confirmed, primary colon cancer, stadium UICC II or III, and histopathological confirmed *R*0-resection. After 7 months of recruitment, an addendum to the study protocol was accepted by the ethics committee for adjusting inclusion criteria (extension of the upper age barrier from <75 years to ≤80 years and inclusion of rectal carcinoma patients).

### 2.3. Intervention

Every patient participated in an individualized endurance-based exercise intervention over 12 months. The aim of the intervention was to increase the patient's PA level up to ≥18 MET*∗*h/week [4] within the first 12 weeks and to maintain this PA level during the intervention period. One MET is equivalent to resting energy expenditure. Multiple METs express exercise intensities that are performed over a certain time, e.g., hours per week. Based on the results summarized in [10], we decided to define 18 MET*∗*h/week as the target PA level.

The exercise intervention consisted of both supervised and home-based training sessions (STS and HTS). Frequency of STS (on average 45 sessions) decreased over the intervention period while frequency of HTS (at least 120) increased. STS was consecutively reduced: during the first 3 months, STS took place twice a week (months 4–6: once a week; months 7–9: every other week), and during months 10–12, STS took place monthly. HTS started with twice a week for the first three months, to 2–3 times in months 4–9, to 3–4 times in months 10–12. For both STS and HTS, during the first six months of training, the intensity was in the range of 60–70% of the peak oxygen consumption (peak VO_2_). In months 7–9, the intensity varied through the use of the interval method between 60 and 80% of peak VO_2_ in order to achieve an increase in physical exercise capacity. In the last three months, the intensity range was increased to 70–80% of peak VO_2_. Each training session lasted 60 minutes and included a 10-minute warm-up (50–60% of peak VO_2_) and a 5-minute cool-down at low intensity.

Due to the changing performance status of CRC patients, especially during chemotherapy, intermittent training was performed at the beginning of the training or as needed (for example, 5-6 minutes of exercise followed by 1-2 minutes of rest). The load phases were then gradually prolonged, and the rest periods were shortened accordingly. As soon as possible, the transition was made to the continuous method. In the further course, the application of the interval method was possible to increase the intensity, e.g., by interval runs with an alternation between intensive walking and light jogging. STS consisted of treadmill running, bicycle-ergometry, or elliptical. HTS consisted of multiple different activities. During the first 3 months, sports such as hiking, walking, Nordic walking, and cycling were practiced. In the further course, other sports were added depending on the physical capacity and preferences of the study participants (e.g., modified ball games, exercise ball games, movement and holding exercises from the areas of Tai-Chi, Qigong, or Yoga), in order to diversify the training. Alternatively, dynamic strength training was performed. However, the focus was on aerobic endurance training.

To optimize patient compliance, STS were free of charge and conducted at licensed rehabilitation centers close to participants' homes. Fixed training times were offered so that the patients could meet each other during training. Every patient obtained a heart rate monitor to control training intensity.

### 2.4. Attendance

The aim was to reach an attendance rate of ≥70%. Attendance was specified as the participation in ≥70% of calculated training sessions (i.e., in at least 116 sessions over 12 months). The patients recorded their daily activities (frequency, modality of training, average heart rate, duration) in paper-based training diaries, which were returned to the trial center every fourth month. The PA reported in the training diaries was transformed into MET*∗*h/week to monitor training progress and calculate attendance rates based on the Compendium of Physical Activities by Ainsworth et al. [4].

### 2.5. Dropouts

Dropouts were defined as patients who did not complete the final study visit. They were continuously documented, including an assessment of the reasons for study withdrawal.

### 2.6. Clinical Examinations and Questionnaires

There were three study visits for each patient (baseline (*V*1), 6 (*V*2), and 12 months (*V*3) after baseline). All visits contained anamnesis, clinical examination, and anthropometric measurements. The psychosocial strain of patients was assessed by questionnaires (*V*1–*V*3): The European Organization for Research and Treatment of Cancer (EORTC), Quality of Life Questionnaire C30 (QLQ-C30) [11] including the colorectal module, the EORTC QLQ Fatigue-Module (FA-13) [12], the Hospital Anxiety and Depression Scale (HADS) [13, 14]. A patient satisfaction questionnaire was handed out at the final study visit including the questions “How high was your motivation for training (a) at the rehabilitation center and (b) at home?” The response options were classified into five levels from very low (0) to very high (4). Cardiopulmonary fitness was determined by peak VO_2_ and assessed by cardiopulmonary exercise testing (CPX) at *V*1 and *V*3. CPX was performed on a bicycle, with a loading scheme of 10/10/1 for very poorly performing patients or 25/25/3 for more powerful patients. That is, the load started at 10 or 25 watts, and there was an increase of 10 or 25 watts every minute or every 3 minutes, respectively, until the maximum workload was reached with the target of exhausting them with a respiratory exchange ratio (RER) > 1.05. The heart rates were documented at the respective intensity increases. On the basis of the maximum oxygen uptake during testing (peak VO_2_) and the heart rates determined during the testing, the patient could be given the optimal training pulse range. For aerobic endurance training, this corresponds to the heart rates determined at 60–80% of the peak VO_2_.

### 2.7. Assessment of Physical Activity

Leisure time PA (LTPA) was measured via the International Physical Activity Questionnaire (IPAQ) (*V*1–*V*3). The IPAQ guidelines were followed including truncation rules to avoid the misclassification of extreme values [15]. If weekly hours for walking, moderate, or vigorous intensity LTPA exceeded 180 minutes for each category, it was truncated to a maximum of 180 minutes per week for each category. Besides this subjective measurement, the AiperMotion440 accelerometers were used to objectively assess PA level. The device was handed out to the patients at the study visits *V*1–*V*3. Participants were instructed to wear it for a period of ten consecutive days on an elastic belt placed at the height of the iliac crest of the nonparetic side, except when sleeping at night, showering, or doing any water-based activity. Seven days were included in the evaluation (minus the first two days and the last day). Patients with less than four valid days were excluded from the analysis. Date of birth, body weight and height, date and time were entered into the device to determine the evaluation period as well as the metabolic rate. Furthermore, MET*∗*h/week was calculated on the basis of training diaries based on the Compendium of Physical Activities by Ainsworth et al. [4] considering months 4–12 (months 1–3: initiation phase for reaching 18 MET*∗*h/week). Lost training diaries were defined as missing values. Incomplete documentation was calculated as no PA (0 MET*∗*h/week).

### 2.8. Safety

All (serious) adverse events ((S)AEs) that occurred during the study were documented in the case report forms by study investigators, regardless of their causal relationship to the intervention. They were recorded continuously and had to be reported back to the study center by using safety report forms. Furthermore, the patients were asked by physicians about their medical history as part of a standardized anamnesis during all study visits with regard to the occurrence of diseases. AEs were defined as diseases, signs of disease, or symptoms that occur or worsen after the inclusion of the patient in the study. SAEs included patient death, life-threatening events, events that cause permanent or significant damage to health or that require hospitalization or prolong hospital stay or that result in permanent disability (inability to work/earn), and secondary malignancies. The causal association of the AE with the intervention (physical training) was given if the event occurred during a reasonable period of time after intervention (within 48 hours), could not be explained by the patient's clinical condition, the environment, toxic factors, or other therapeutic interventions, could be attributed to known adverse effects of the intervention, improved or resolved after discontinuation of the intervention, or recurred after the resumption of the intervention.

### 2.9. Statistical Analyses

Data analysis was performed using IBM SPSS 23.0 (IBM Corporation, Armonk, NY, USA) and *R* version 3.4.4 (R Foundation for Statistical Computing, Vienna, Austria). For quantitative data means, standard deviations, minima and maxima or, for skewed data, medians, and interquartile ranges (presented as first-third quartile) are given. Absolute and relative frequencies are presented for categorical variables. Paired or unpaired *t*-tests were performed to compare mean changes in quantitative data or assess mean differences between groups. Distributions of leisure-time PA and sitting time per day were compared between groups using Mann–Whitney *U* tests and between timepoints (paired data) using Wilcoxon signed rank tests, as data were not normally distributed. For the comparison of distributions of categorical variables between groups, Fisher's exact test was used. All tests were performed two-sided, and a significance level of 5% was used.

## 3. Results

### 3.1. Patient Characteristics

The mean age of the 50 participants (23 female) was 59.0 ± 10.3, ranging from 32–80 years. All patients were in the WHO performance status 0 or 1 [16]. The majority of the patients (*n* = 35) were recruited within 3 months after surgery. Six patients were recruited over a period of more than 6 months after surgery. The mean duration between surgery to study entry and first training was 86.8 ± 54.7 (22–226) days and 102.9 ± 56.8 (41–257) days, respectively. 34 of the study participants had colon cancer (44% UICC III), 15 patients had rectal cancer (87% UICC III), and one patient was diagnosed with both colon and rectal cancer (UICC II). There were no statistically significant differences between patients who received adjuvant chemotherapy (*n* = 30; 60%) and patients who did not receive their changes in PA levels and peak VO_2_ from baseline to 12 months, nor in the frequencies of compliance or reaching the 18 MET^∗^h/week from week 13 onwards. There were no statistically significant differences in baseline characteristics between sexes.

### 3.2. Recruitment

**Figure 1:**
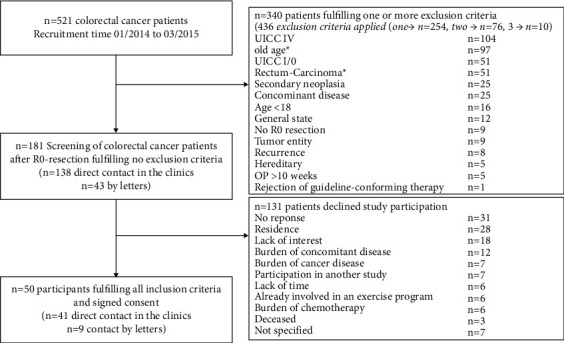
Flowchart of participant recruitment 01/2014–03/2015. ^∗^before study addendum

Participant recruitment took place from 01/2014 to 03/2015. 50 patients were recruited within 15 months out of 521 CRC patients who were screened for eligibility. Leading causes for exclusion were UICC IV stadium (20%), UICC I/0 stadium (10%), and age (19%, before the study addendum). 131 of 181 eligible patients declined study participation (recruitment rate of 28%), of whom 62% (*n* = 80) were male and 38% (*n* = 50) were female (1 was excluded from the calculation because of missing information). Decliners were statistically significantly older compared to the included patients (66.2 ± 9.8 vs. 58.9 ± 10.3 years, *p* < 0.001). The leading cause for declining was a too long distance from the patients' residence to the study center (see Figure 1).

### 3.3. Dropouts

The dropout rate was 18% (*n* = 9; *n* = 6 before an intermediate visit, *n* = 3 before the final visit). Dropouts were due to lack of motivation (*n* = 3), occupational reasons (*n* = 2), lack of time (*n* = 1), chemotherapeutical side effects (i.e. polyneuropathy, foot pain) (*n* = 1), and cancer recurrence (*n* = 1). Contact with one participant was lost. There were no statistically significant differences between dropouts and participants who completed the study for the variables age, sex, stage of disease, adjuvant therapy, and distance to training centers.

### 3.4. Attendance

**Figure 2:**
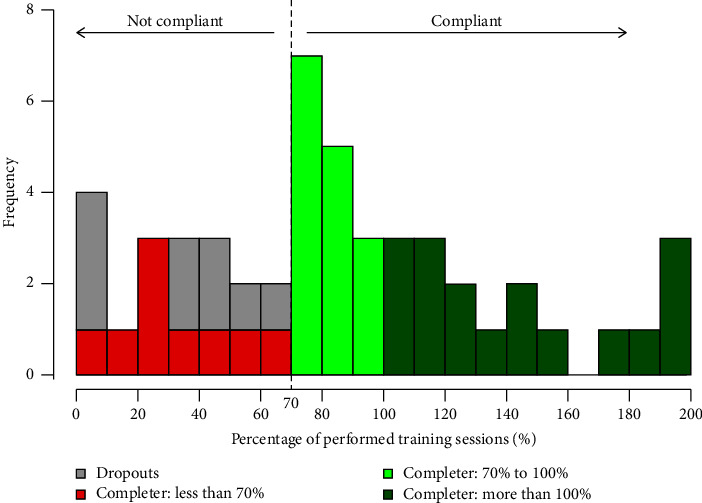
Number of patients considering the percentage of performed training sessions.

The mean time patients spent within the intervention was 44 ± 16 weeks. Attendance (participation of at least 70% of training sessions) was reached by 64% of the participants including the 9 dropouts (*n* = 32 of *n* = 50). Excluding the 9 dropouts, attendance was 78% (Figure 2). Patient's motivation for STS was indicated as very high by 44.4% and as high by 36.1%, while this was 13.2% and 50.0% for HTS.

**Figure 3:**
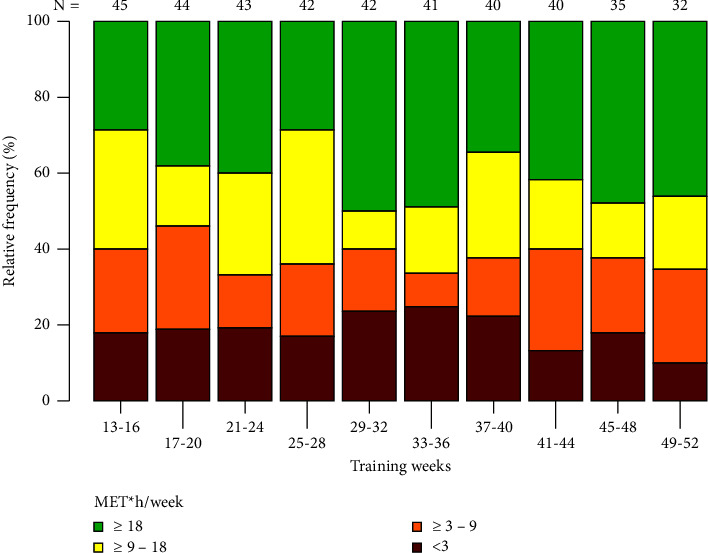
Categories of MET-hours per week reached by study participants during the intervention.

19 patients (38%) reached >10 MET*∗*h/week in more than 70% of training weeks 13–52, and 6 patients (12%) maintained a level of ≥18 MET*∗*h/week over >70% of training weeks 13–52.  Figure 3 shows the percentages of patients reaching <3, ≥3–<9, ≥9–<18, and ≥18 MET*∗*h/week for each month (weeks 13–52). The mean PA level for all training weeks 13–52 was 14.4 ± 12.8 MET*∗*h/week. For 411 of the scheduled training weeks (21%), training diaries were missing. The differences in baseline characteristics in compliant versus noncompliant patients are shown in Table 1.

Concerning safety, nine SAEs (*n* = 2 scar fracture, *n* = 1 achilles tendon rupture, *n* = 1 bridenileus, *n* = 1 cancer recurrence, *n* = 1 abdominal wall hernia, *n* = 1 polypectomy, *n* = 1 metastases, *n* = 1 new occurrence of Morbus Parkinson) and 19 AEs (in 16 patients) occurred (*n* = 1 pain on the right foot, *n* = 1 lumboischialgia, *n* = 1 herniated disc, *n* = 1 knee pain, *n* = 1 renal stones) including side effects from chemotherapy (*n* = 9 polyneuropathy, *n* = 5 nausea, diarrhea, and/or vertigo). All (S)AEs were assessed by the investigator as not causally related to the intervention.

**Figure 4:**
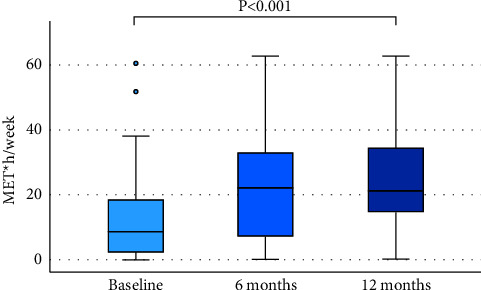
Leisure-time physical activity at baseline, 6 months and 12 months.

Changes in the patient characteristics over the course of the intervention are shown in Figure 4 and Table 2.

## 4. Discussion

The F-PROTECT study has shown that the present intervention involving a combination of supervised and home-based exercise training in postsurgical CRC patients was not feasible according to the defined criteria. The recruitment period of the targeted 50 patients exceeded the goal of 12 months by 3 months, which is also reflected in the low recruitment rate of 28% (50/181). The attendance to STS was 6% below the 70% to be achieved. Furthermore, only 12% of participants reached the intended ≥18 MET*∗*h/week of exercise in ≥70% of training weeks up to week 13.

### 4.1. Recruitment

A recent systematic review and meta-analysis found an overall recruitment rate of 38% based on 16 exercise intervention trials in CRC patients. Importantly, recruitment rates differed according to the duration of the intervention (62% for <12 weeks; 33% for ≥12 weeks). In addition, they found notable differences in the timing of treatment initiation. The recruitment rate in studies that started the intervention after treatment was 28%, while it was 33% during treatment and 88% before treatment [8]. In our study, the majority of patients were recruited during their inpatient stay with ongoing cancer therapy. The median time from surgery to study inclusion was 9.5 weeks. The short time span from cancer diagnosis and surgery to study inclusion, as well as the stress from chemotherapy, may have discouraged patients from participating in an exercise intervention study. However, Brown et al. have shown that recruitment was most successful during a more restrictive phase of recruitment (completed surgical resection and adjuvant chemotherapy ≤24 months before entering the study compared to ≤36 months). There is probably a window of time relatively close to therapy when patients are particularly motivated to make lifestyle changes [17].

On average, we enrolled 3.3 patients at 10 departments over 15 months. A further study in CRC patients investigating a home-based interval-walking intervention delivered by a smart-phone application that has also applied in-clinic recruitment achieved a number of 2.4 patients (39 over a period of 16 months) per month at 5 oncology departments [18]. A way to increase the recruitment numbers could be the use of population-based cancer registries along the lines of Brown et al., with which they were able to contact more than 1.500 potential candidates via letters. The screening was conducted via telephone interview. Anyhow, they enrolled 39 patients over 7 months (5.4 patients per month) at a single institution [17]. Furthermore, it might be useful for future trials to not exclude patients based on their metrics but to also account for the study staff's ability to motivate patients to engage in tertiary preventive measures. Accompanying, the final selection of patients could be done to a higher extent by the main trial centers with an expertise in sports medicine. The possible lack of sports medicine knowledge of the clinicians on-site at the surgical clinics could have led to overcaution and reluctance to propose patients for the study. Sports medicine departments have experience in evaluating the fitness of patients independently of comorbidities or old age factors that are not necessarily accompanied by reduced ability to exercise.

A far distance to the trial center or to the training locations was identified as the main reason for the refusal of study participation in previous exercise intervention studies for cancer patients [6, 19–21]. We tried to prevent this problem and offered different training locations located near the patients' homes. The distance to travel to the trial center was still considered as a key reason for refusal. Therefore, the number of visits to the trial center should be limited in the future in order to increase recruitment and compliance.

### 4.2. Attendance and Dropouts

In the meta-analysis of Singh et al., a median adherence of 85% (42–91%) was reported among 19 exercise intervention trials with intervention durations ranging between 1 week and 6 months [8]. To compare other feasibility studies, Sellar et al. achieved attendance rates of more than 90% with 27 of 29 CRC patients fulfilling ≥80% of STS within an intervention period of 12 weeks [20]. Bourke et al. had attendance rates of 90% during the 12 weeks of supervised and home training [22]. Our attendance rate is relatively low compared to other studies but seems to be realistic when taking the long training period of 12 months into account. Furthermore, supervision was reduced because the goal was to enable patients to maintain PA independently. In the comparable CHALLENGE trial, 83% of participants completed the mandatory STS in the first six months and 68% completed the optional STS in the second six months [23]. Based on the high level of patient's satisfaction with STS, consideration should be given to maintain a mix of supervised and home training for future studies. This approach is supported by the overall results of a current meta-analysis showing a significant effect on the quality of life and functional capacity regarding supervised or mixed intervention (6 studies) while no significant effect was observed for home-based intervention (5 studies) [24].

The meta-analysis by Singh et al. reported a median withdrawal rate of 12% (0–22%) compared to 18% in the F-PROTECT study. The feasibility study of the CHALLENGE trial reported a 10% dropout rate. Importantly, the trial included specific behavior change techniques that may have improved motivation to continue participation in the study [23].

The comparison between compliant versus noncompliant patients in our study indicates that patients with advanced stages of cancer, low overall performance status, and low PA status need to achieve special attention in exercise intervention trials.

### 4.3. Changes in Physical Activity

The amount of self-reported LTPA increased statistically significant from baseline to 12 months, which is in line with other PA intervention studies [21, 23, 25, 26]. However, results of objectively measured PA revealed no statistically significant changes in PA level nor metabolic rate. Furthermore, the aim of the PA intervention to reach and maintain a threshold of ≥18 MET*∗*h/week was not successfully realized, as only 6 participants achieved ≥18 MET*∗*h/week in ≥70% of training weeks. Sellar et al. were able to achieve a PA level of ≥18 MET*∗*h/week but acknowledged that further studies need to investigate whether this can be maintained over a longer period of time because their intervention periods were only 12 weeks [20, 25]. Although the lack of documentation was counted as 0 MET*∗*h/week for calculation purposes, which may have resulted in an underestimation of PA volume when training diaries were taken into account, the results of the F-PROTECT study suggest that this extensive PA volume is not realistically achievable for most CRC patients over a long time period. However, the question arises whether a lower PA level might be sufficient, also considering current recommendations of 150 min/week of moderate intensity PA, which corresponds to 10 MET*∗*h/week. The systematic review and meta-analysis by Schmid and Leitzmann showed that there seems to be a dose-response effect concerning PA and mortality in CRC patients. Each 10 MET*∗*h/week increase in PA after diagnosis was associated with a 28% lower risk of all-cause mortality [10]. Similarly, in a subanalysis by Meyerhardt et al., patients who increased their PA and those who were consistently active of at least 9 MET*∗*h/week had an improvement in CRC-specific mortality compared with relatively sedentary patients who had no change in PA [27]. Accordingly, the goal in the CHALLENGE trial is to increase leisure-time PA by ≥ 10 MET*∗*h/week from baseline during the first 6 months and to maintain this change for 3 years. This was successfully achieved in their one-year feasibility study [23]. Future RCTs need to investigate lower PA doses associated with survival benefits and transferability to clinical practice.

Interestingly, there was an increase in BMI as well as in waist circumference and body fat which is in line with the results of a current meta-analysis including 7 RCTs and 803 CRC patients, which has shown no significant within groups effects of PA interventions for BMI [3]. This may be interpreted in the context of the timing of diagnosis and cancer treatment. An observational study in 485 stage II/III CRC patients has shown that body weight decreased during surgery and increased during and after chemotherapy [28]. However, as we do not have information on dietary intake and due to the lack of a control group, we can only speculate on these findings. Importantly, our results reveal that an increase in peak VO_2_ was statistically significantly correlated with a decrease/less increase in waist circumference (*r* = −0.413; *p* = 0.011), which highlights the importance of increasing fitness to improve the prognosis in this patient group.

### 4.4. Safety

Twenty-eight(S)AEs occurred during the study participation. Even though they were assessed as not training-related which is in accordance with the assessment of the safety of exercise in previous interventional trials [8], the high incidence of complications in this patient group is certainly a major barrier to exercise intervention studies.

### 4.5. Study Strengths and Limitations

The F-PROTECT study examined a much longer intervention period than most previous feasibility studies in this population, providing important information about the feasibility of maintaining relatively high PA volumes over long periods of time. Moreover, a relatively large number of patients were included compared with other feasibility studies of PA in CRC. Physical fitness was measured by peak VO_2_, which is considered the gold standard for measuring maximal aerobic capacity.

An important limitation is the lack of a control group. However, the aim of this study was to demonstrate the feasibility and not the superiority of the intervention. Thus, our results cannot determine causality.

Furthermore, selection bias must be considered (patients willing to participate may be healthier/fitter). As can be seen from the results, the study participants were relatively active at baseline. However, the cardiorespiratory fitness was relatively low and comparable to other studies involving CRC patients [9, 29]. Nevertheless, our study population may represent a high-selective group, which does not necessarily reflect the patient strata presenting for treatment. However, attendance to protocols was difficult, probably due to the high amount of required exercise. Additionally, feasibility and attendance rates are critically dependent on multiple factors, such as study setup, patient's preference of exercise type (strenuous vs less strenuous exercise, focusing on different aspects of motoric abilities, etc.) center capabilities, local distances to training centers, or underlying population demographics. Therefore, translating results from this study to other sites has to be done with caution.

## 5. Conclusions

The feasibility of an endurance-based intervention over a 12-month period involving a combination of supervised and home-based training in postsurgical CRC patients could not be confirmed.

Researchers should therefore develop exercise programs specifically tailored to CRC patients that they can maintain over the long term. Furthermore, future RCTs should aim to optimally monitor PA (e.g., telemedical) to allow the calculation of a PA continuum to define a realistic and sufficient threshold for improving prognosis.

## Figures and Tables

**Table 1:** Baseline characteristics in compliant versus noncompliant patients.

Baseline characteristic	Compliant, *n* = 32	Noncompliant, *n* = 18	*p* value^*∗*^
	*n* (%)		
Sex			1.000
Male	17 (53%)	10 (56%)	
Female	15 (47%)	8 (44%)	
Stage of disease			0.018
UICC II	19 (59%)	4 (22%)	
UICC III	13 (41%)	14 (78%)	
Smoking			0.077
Smoker	12 (38%)	12 (67%)	
Nonsmoker	20 (63%)	6 (33%)	
Adjuvant therapy			0.556
Yes	18 (56%)	12 (67%)	
No	14 (44%)	6 (33%)	
Neoadjuvant radio-chemotherapy			0.309
Yes	6 (19%)	6 (33%)	
No	26 (81%)	12 (67%)	
Surgical approach			0.144
Laparoscopy	12 (38%)	11 (61%)	
Laparotomy	20 (63%)	7 (39%)	
Stoma			0.609
Yes	5 (16%)	3 (17%)	
No	27 (84%)	15 (83%)	
Comorbidities (based on ICD-10 codes)			0.735
Yes	25 (78%)	13 (72%)	
No	7 (22%)	5 (28%)	
Metabolism	7/25	2/16	0.459
Circulation	10/22	6/12	1.000
Respiratory	3/29	0/18	0.544
Digestive	9/23	2/16	0.287
Musculoskeletal	2/30	3/15	0.336
Blood	6/26	1/17	0.398
Others	5/27	6/12	0.172
Serious adverse events			0.587
Yes	6 (19%)	3 (17%)	
No	26 (81%)	15 (83%)	
Adverse events			0.074
Yes	13 (41%)	3 (17%)	
No	19 (59%)	15 (83%)	
Distance to the training center			0.771
≤10 km	18 (60%)	9 (50%)	
11–30 km	8 (27%)	6 (33%)	
≥30 km	4 (13%)	3 (17%)	
	Mean ± SD		
Age (years)	59.6 ± 10.1	57.8 ± 10.7	0.554
Body mass index (kg/m^2^)	25.7 ± 4.6	26.6 ± 4.6	0.508
Karnofsky index (%)	90.5 ± 5.1	84.4 ± 7.3	0.015
QoL-global health status (0–100 points)	66.4 ± 19.8	62.0 ± 15.2	0.424
LTPA (MET^*∗*^h/week)^†^	15.4 ± 14.7	5.14 ± 5.4	0.007
PA level^‡^	1.4 ± 0.2	1.4 ± 0.2	0.516
Metabolic rate (kcal)^‡^	554.7 ± 268.8	480.4 ± 290.0	0.520
Sitting time per day (hours)	5.8 ± 3.4	5.3 ± 3.2	0.629
Days between surgery to study entry	83.0 ± 56.8	93.7 ± 51.6	0.510
Days between surgery to first training	101.0 ± 59.1	106.3 ± 53.9	0.755
PeakVO_2_ (ml·min^−1^·kg^−1^)	22.8 ± 6.4	20.7 ± 5.2	0.244

^*∗*^ Fisher's exact test for binary data, Mann–Whitney *U* Test for skewed quantitative data (LTPA, sitting time per day), independent *t*-test for other quantitative data; SD = standard deviation; QoL = quality of life; LTPA = leisure time physical activity; UICC = Union for International Cancer Control. ^†^ measured by IPAQ questionnaire. ^‡^ measured by accelerometers. Peak VO2 = peak oxygen consumption; MET = metabolic equivalent of tasks.

**Table 2:** Patient characteristics from baseline to 12 months.

Characteristic	Baseline			6 months			12 months			*p*-value^*∗*^
	*n*	Mean ± SD	Min–Max	*n*	Mean ± SD	Min–Max	*n*	Mean ± SD	Min–Max	
Body weight (kg)	50	77.6 ± 15.5	48–106	44	81.7 ± 16.1	46–114	41	82.2 ± 15.7	57–116	<0.001
Waist circumference (cm)	49	91.5 ± 13.2	67–117	42	92.4 ± 12.7	70–121	41	95.7 ± 12.9	74–123	0.001
BMI (kg/m^2^)	50	26.0 ± 4.6	20–40	44	27.3 ± 4.9	19–42	41	27.5 ± 4.9	20–44	<0.001
Body fat (%)	49	27.8 ± 8.8	12–49	37	28.2 ± 8.0	17–51	35	29.9 ± 6.9	16–45	0.008
Karnofsky index (%)	29	88.6 ± 6.4	80–100	33	96.1 ± 5.0	90–100	31	94.2 ± 6.3	80–100	0.157
QoL-global health status (0–100 pts)	49	64.8 ± 18.2	33–100	45	66.5 ± 19.4	33–100	37	75.0 ± 15.6	33–100	0.010
QoL-fatigue (0–100 pts)	49	33.1 ± 28.0	0–100	45	27.2 ± 23.6	0–89	37	18.9–18.7	0–78	0.002
Anxiety (HADS 0–21 pts)	48	8.4 ± 2.0	5–12	45	8.2 ± 2.1	5–13	37	7.8 ± 1.8	5–12	0.115
Depression (HADS 0–21 pts)	48	9.8 ± 1.8	6–13	45	9.4 ± 1.7	6–13	37	9.8 ± 1.8	7–14	0.477
LTPA (Met*∗*h/wk)^†^	50	11.7 ± 13.1	0–61	45	20.0 ± 15.7	0–63	40	24.6 ± 16.1	0–63	<0.001
PA level^‡^	26	1.4 ± 0.2	1.0–1.8	22	1.5 ± 0.2	1.0–1.8	28	1.4 ± 0.2	1.0–2.0	0.797
Metabolic rate (kcal)^‡^	26	529.0 ± 272.9	24–1,1105	22	693.5 ± 255.8	48–1337	28	730.8 ± 765.6	4–4332	0.328
Rel. peak VO_2_ (ml·min^−1^·kg^−1^)	49	22.0 ± 6.0	9.6–36.0	—	—	—	39	24.3 ± 6.7	13–38	0.002
Abs. peak VO_2_ (ml·min^−1^)	49	1,708.8 ± 562.3	857–3,389	—	—	—	39	1,964.9 ± 563.0	995–3,352	<0.001
Watt_@VT1_	49	59.4 ± 41.0	3–200	—	—	—	38	80.7 ± 45.6	8–200	<0.001
Watt_max_	49	133.6 ± 44.5	70–250	—	—	—	38	156.4 ± 53.1	75–250	<0.001

^*∗*^ Paired *t* test (changes from baseline to 12 months, based on individuals with both baseline and 12 months data). SD = standard deviation, Min = minimum, Max = maximum; QoL = quality of life; LTPA = leisure time physical activity. ^†^measured by IPAQ questionnaire. ^‡^measured by accelerometers.

## Data Availability

The data that support the findings of this study are available on request from the corresponding author.

## Abbreviations

AE: Adverse event
CPX: Cardiopulmonary exercise testing
CRC: Colorectal cancer
EORTC: The European organization for research and treatment of cancer
FA-13: Fatigue module
FBK: The German “*fragebogen zur belastung von krebspatienten'*”
F-PROTECT: Feasibility of the potential role of tertiary prevention by exercise in colorectal cancer therapy
HADS: Hospital anxiety and depression scale
HTS: Home-based training sessions
IPAQ: International physical activity questionnaire
LTPA: Leisure time physical activity
MET: Metabolic equivalent of tasks
PA: Physical activity
peak VO_2_: Peak oxygen consumption
QLQ-C30: Quality of life questionnaire including the colorectal module
RCT: Randomized, controlled trial
SAE: Serious adverse event
STS: Supervised training session
UICC: Union for International Cancer Control.
